# Influenza A(H1N1)pdm09 virus infection in marine mammals in California

**DOI:** 10.1038/emi.2013.40

**Published:** 2013-06-26

**Authors:** Walter M Boyce, Ignacio Mena, Pamela K Yochem, Frances MD Gulland, Adolfo García-Sastre, Noelia Moreno, Daniel R Perez, Ana S Gonzalez-Reiche, Brent S Stewart

**Affiliations:** 1 Department of Pathology, Microbiology and Immunology, School of Veterinary Medicine, University of California, Davis, CA 95616, USA; 2 Department of Microbiology, Icahn School of Medicine at Mount Sinai, NY 10029, USA; 3 Physiology and Ocean Health Program, Hubbs-SeaWorld Research Institute, San Diego, CA 92109, USA; 4 The Marine Mammal Center, Sausalito, CA 94965, USA; 5 Department of Veterinary Medicine, University of Maryland, College Park, MD 20742, USA; 6 Ecology Program, Hubbs-SeaWorld Research Institute, San Diego, CA 92109, USA

## 

**Dear Editor,**

In a recent report, some of the coauthors of this letter documented the isolation of influenza A(H1N1)pdm09 from northern elephant seals (NES, *Mirounga angustirostris*) in California in 2010.^1^ The two virus sequences were most similar to a December 2009 human isolate from San Diego, California, raising many questions as to the timing and extent of virus transmission among marine mammals. This letter presents new data showing that three co-occurring marine mammal species were exposed to A(H1N1)pdm09 as early as 2009, and that widespread epidemic transmission subsequently occurred among NES, but not harbor seals (HS, *Phoca vitulina*) or California sea lions (CSL, *Zalophus californianus*).

Over 300 000 seals and sea lions live in the eastern North Pacific Ocean and haul out to molt and give birth along the mainland and island coastlines of California. We obtained sera from the three most abundant pinniped species in this region, and tested them for the presence of A(H1N1)pdm09-specific antibodies by hemagluttination inhibition (HI). We chose samples from NES (*n*=222), CSL (*n*=183) and HS (*n*=140) belonging to populations that spanned the US coastline from Mexico to Oregon to determine if exposure was widespread or limited to the small region and population examined previously.^1^ We also selected samples that covered the period from 2009–2011 to examine the timing of A(H1N1)pdm09 emergence relative to its first reported detection in humans (2009) and NES (2010). Samples included free-ranging animals that had minimal human contact and were sampled on the Channel Islands off the coast of southern California, and animals that stranded along the central and northern California coastline and were hospitalized at The Marine Mammal Center.

In addition to determining the timing, host range, and geographic extent of A(H1N1)pdm09 exposure, we investigated whether or not A(H1N1)pdm09 transmission was endemic among NES. We focused surveillance efforts on females and their pups on the Southern California Channel Islands since 75% of the extant NES population of >170 000 seals breed and haul out on these islands.^2^ We collected nasal and rectal swabs and serum samples from 20 nursing pups and their mothers at San Nicolas Island in January 2012 when the pups were 2∼4 weeks old, and resampled the same 20 pups a month later in February after they were weaned. We sampled another 15 females and their nursing pups a single time at San Miguel Island in January 2012. Swabs collected from each animal were placed in viral transport media and tested by matrix real-time polymerase chain reaction and inoculation in embryonating chicken eggs, and sera were tested by HI.

This study shows for the first time that A(H1N1)pdm09 emerged in marine mammals along the California coast as early as 2009, and that multiple species were exposed and produced A(H1N1)pdm09-specific antibodies (Table 1). Seropositive NES and HS were first detected in 2009, and the first seropositive CSL were sampled in 2010. In contrast to HS and CSL, NES appeared to be uniquely susceptible to infection and epidemic transmission, with widespread exposure occurring in populations and regions far beyond where virus was isolated in 2010.^1^ By 2011, 48% of NES sampled along the 1000 km California coastline had HI titers ≥40, and 54% of adult females and 63% of nursing pups at the Channel Islands were seropositive in 2012. This high percentage of HI-positive results is not due to intrinsic high background in NES serum, since samples in this and the previous study were clearly negative when tested against other human influenza strains.^1^

In 2012 on San Nicolas Island, there was a trend among all seropositive NES pups for titers to increase with age (i.e., pre-weaning to post-weaning), and 30% (6/20) of the pups sampled twice showed a four-fold titer increase over a one-month period. This four-fold increase is consistent with an anamnestic antibody response to acute infection, but could also be explained by nursing pups gradually accumulating maternal antibodies.^3^ Positive/negative results were largely correlated between females and pups, further supporting the hypothesis of maternal antibody transfer. No evidence of influenza A viruses or viral RNA were detected in nasal or rectal swabs, thus it appears unlikely that A(H1N1)pdm09 transmission occurred at the NES colony in 2012.

Although exposure to A(H1N1)pdm09 was detected in CSL and HS, there was no evidence of widespread transmission or maternal antibody transfer comparable to that seen in NES. Four of 60 stranded HS pups hospitalized at The Marine Mammal Center in northern California were seropositive in 2009 and 2010, but no positive adults or pups were detected in 2011 (*n*=50). Likewise, seven adult and sub-adult CSL were seropositive in 2010 and 2011, but no pups were seropositive from 2009–2011 (*n*=90).

We hypothesize that reduced genetic variability and other species-specific attributes may influence the susceptibility of NES to A(H1N1)pdm09 relative to HS, CSL and other pinniped species. NES underwent a severe demographic and genetic bottleneck, recovering from an estimated total population of <100 seals in the 1800's to >170 000 animals today, and genetic studies have shown a virtual lack of variability at nuclear and mitochondrial loci.^4,5,6^ A recent study suggested that human influenza A viruses attach poorly to the respiratory epithelium of HS and other marine mammals, but no data are available for NES.^7^

Marine mammals, especially true seals (Phocidae) like NES, may play a role similar to swine as reservoirs and mixing hosts for avian and mammalian viruses.^8^ The recent outbreak of avian-origin H3N8 in Atlantic harbor seals (*Phoca vitulina*) along the US east coast, and reports of influenza infection and susceptibility in other seal species elsewhere, clearly support this hypothesis.^9,10,11,12,13,14,15^ However, the sudden appearance and rapid geographic spread of a highly contagious influenza A virus in both humans and marine mammals is unprecedented. The potential for marine mammals to be co-infected with human and avian origin viruses creates troubling opportunities for reassortment and the emergence of new virulent genotypes.

## Figures and Tables

**Table 1:** Antibodies to A(H1N1)pdm09 in seals and sea lions in California, USA, 2009–2012

	% HI positive			
	Northern elephant seals	Harbor seals^3^	California sea lions^4^	Range HI titers
2009^1^	5 (2/37)	8 (3/40)	0 (0/80)	<10 to 160
2010^1^	30 (11/37)	2 (1/50)	7 (4/55)	<10 to 640
2011^1^	48 (29/60)	0 (0/50)	6 (3/48)	<10 to 640
2012				
Adult females^2^	54 (19/35)	-	-	<10 to 160
Pups 1st sample^2^	63 (22/35)	-	-	<10 to 320
Pups 2nd sample^2^	75 (15/20)	-	-	<10 to 320

Sera tested by HI at dilutions of 1/10, 1/20, 1/40, 1/80, 1/160, 1/320, 1/640. Titers ≥40 were considered positive.

1 Free-ranging and stranded adults and juveniles in northern and southern California.

2 Free-ranging adults and pups at the Southern California Channel Islands.

3 Seropositive harbor seals were stranded weaned pups from northern and central California with HI titers of 40 to 160.

4 Seropositive California sea lions were stranded and free-ranging subadults and adults from northern and southern California with HI titers of 40 to 80.
